# Mental health support through transcendental cinema. “Mulholland Drive” By D. Lynch as an example of a mindfulness meditation session

**DOI:** 10.1192/j.eurpsy.2024.1375

**Published:** 2024-08-27

**Authors:** J. K. Nowocień, N. Szejko

**Affiliations:** Department of Medical Ethics and Palliative Medicine, Medical University of Warsaw, Warsaw, Poland

## Abstract

**Introduction:**

Regular practice of mindfulness has proven effectiveness in the treatment of affective disorders (Cash and Whittingham, 2010), increases the level of satisfaction in life (Brown and Ryan, 2003), as well as the level of self-esteem (Rasmussen & Pidgeon, 2010). Using “Mulholland Drive” (2001) by David Lynch as an example, we will prove that transcendental cinema, through the unique slow character, the means of expression used and the emphasis placed on the metaphysical experiences of the characters, in which D. Lynch forces us to participate, makes the screening similar to a meditation session.

**Objectives:**

The aim of this work is to indicate a new direction of research, linking cinema with psychoanalysis, philosophy and psychiatry and proving that a screening of transcendental cinema can be treated as a mindfulness meditation session.

**Methods:**

This work is based on the film “Mulholland Drive” by D. Lynch and the understanding of transcendental cinema according to Paul Schrader. Using J. Kabat Zinn’s scientific publications, we analyze cinema in terms of a meditation session and using the approach of first generation analysts (S. Freud, C. Gustav Jung, S. Spielrein) in terms of a therapeutic process based on psychoanalysis.

**Results:**

“Mulholland Drive” subjects the protagonist to a therapy session: the woman lives guided by the unconscious, a dream that seems real and finally at the end she reaches her own true self. On screen, she undergoes successfully the therapeutic process. The transcendental cinema focuses on metaphysical sensations, has elongated scenes, creates *dead time*, viewer remains in the frame even when the character comes out of it, and strives for *kenosis* - the reduction of sensory experience. All of these qualities are crucial in mindfuless: focusing on emotions and feelings experienced in the moment, non-judgmental and calmly concentrating on single stimuli. This similarity allows us to treat a transcendental film show in the category of a meditation session.

**Conclusions:**

Recognizing the similarity between the philosophy of mindfulness and transcendental cinema allows us to conclude that a film screening enriches us not only with knowledge about disorders and the therapeutic process, but is in itself a supportive element for mental health. Our work is the first to analyze cinema in the context of mindfulness meditation. In our opinion, culture should be more widely analyzed as a tool to support mental health and the development of one’s own identity.

**Disclosure of Interest:**

None Declared

